# Selective bioorthogonal probe for *N*-glycan hybrid structures

**DOI:** 10.21203/rs.3.rs-3093724/v1

**Published:** 2023-08-01

**Authors:** Mana Mohan Mukherjee, Lara K. Abramowitz, Bhoj Kumar, Parastoo Azadi, John A Hanover

**Affiliations:** 1 Laboratory of Cell and Molecular Biology, NIDDK, National Institutes of Health, Bethesda, MD; 2 Complex Carbohydrate Research Center, The University of Georgia, 315 Riverbend Road, Athens, GA 30602, USA

**Keywords:** Bioorthogonal chemistry, 1,3-Pr_2_-6-OTs GlcNAlk, Enzymatic labeling, Hybrid *N*-Glycan, Fibrillarin nuclear protein

## Abstract

Metabolic incorporation of chemically tagged monosaccharides is a facile means of labelling cellular glycoprotein and glycolipids. Yet, since the monosaccharide precursors are often shared by several pathways, selectivity has been difficult to attain. For example, *N*-linked glycosylation is a chemically complex, and ubiquitous post translational modification with three distinct classes of GlcNAc-containing *N*-glycan structures: oligomannose, hybrid, and complex. Here we describe synthesis of 1,3-Pr_2_-6-OTs GlcNAlk as a next generation metabolic chemical reporter (MCR) for the specific labeling of hybrid *N*-glycan structures. We first developed a general strategy for defining the selectivity of labelling with chemically tagged monosaccharides. We then applied this approach to establish that 1,3-Pr_2_-6-OTs GlcNAlk is specifically incorporated into hybrid *N*-glycans. Using this MCR as a detection tool, we carried out imaging experiments to define the intracellular localization and trafficking of target proteins bearing hybrid *N*-glycan structures.

## Introduction.

Glycans are present in cells as covalent attachments to biomolecules like proteins (glycoproteins), peptides (peptidoglycans) and lipids (glycolipids). It is estimated that 50–70% of all proteins in nature are glycosylated,^1^ and many of the glycosylation events have functional implications. The glycans are very diverse in structure. The two major glycosidic linkages to proteins involve either oxygen in the side chain of serine or threonine (*O*-linked glycans) or nitrogen in the side chain of asparagine (*N*-linked glycans). Additionally, proteins can be attached to the cellular membrane by a linkage between the carboxyl-terminal group and a glycophosphatidylinositol (GPI) anchor. Gangliosides containing sialic acid and other glycolipids are a type of lipid glycosylation. Intracellular proteins have also been shown to be modified by O-GlcNAc,^2^ the *O*-GlcNAc transferase (OGT) uses the uridine diphosphate sugar donor, UDP-GlcNAc, to add a single monosaccharide GlcNAc to serine/threonines of intracellular proteins.^3^ *O*-GlcNAc can be subsequently removed by *O*-GlcNAcase (OGA), rendering *O*-GlcNAcylation dynamic.^4^ On the other hand, *N*-linked glycosylation is a process in which the endoplasmic reticulum (ER) membrane resident enzyme oligosaccharyltransferase (OST) complex mediates an *en bloc* transfer of the oligosaccharide portion of the lipid-linked oligosaccharide (Dol-PP-GlcNAc_2_Man_9_Glc_3_) onto the acceptor asparagine of nascent proteins, defined by the consensus sequence Asn-X-Thr/Ser (X ≠ Pro). The protein-linked glycan structure is then further processed and chemically derivatized.^5^ Protein modifications by *N*- or *O*-glycans can modulate the protein’s biophysical properties and consequently regulate function.^6^

*N*-glycans that are covalently linked to proteins are involved in a myriad of biological processes, including modification of protein folding,^7^ ER quality control and trafficking to the cell surface,^8^ modulation of protein orientation and stability, oligomerization and aggregation,^9^ modulation of enzyme activity,^10^ host cell-surface interactions,^11^ viral infection with such viruses like HIV,^12^ influenza,^13^ and SARS-CoV-2^14, 15^ and recently described as a modification of small RNA.^16^ There are three types of *N*-glycan structures, oligomannose, hybrid, and complex structures. Oligomannose *N*-glycan structure (Glc_3_Man_9_GlcNAc2-Asp) is cleaved to the Man_5_GlcNAc_2_-Asp processing hybrid and further subjected to a complex series of branching, trimming and extension reactions to generate complex type of N-glycans.^17^ The biological roles of complex and oligomannose glycans have been the subject of numerous investigations. However, specific or differential roles of hybrid *N*-glycans are currently unclear.^18^ Until defined specific roles are identified, these hybrid *N*-glycan structures are considered as ‘intermediates’ on the pathway between the oligomannose glycans formed in the ER to the complex glycan structures produced in the Golgi.^19^ Currently, there are no available tool to specifically detect and study hybrid type *N*-glycans. The biological importance of dissecting the physiological role of the various glycan structures is underscored by the fact that hybrid/complex *N*-glycans are essential to embryonic development, as *N*-acetylglucosaminyltransferase (GlcNAcT-I, MGAT1) deficient mice are embryonic lethal.^20^

Recent efforts towards development of methods to study biomolecules in their native environment have employed the tools of bioorthogonal chemistry.^21, 22^ Bioorthogonal chemistry typically involves a two-stage strategy; first the enzymatic incorporation of the metabolic chemical reporter (MCR), followed by bioorthogonal labeling of the MCR with fluorophores or affinity tags.^23^ To date, the most commonly used bioorthogonal reaction is the “click” reaction between metabolically incorporated azide or alkyne derivative and its counter fluorophore.^24, 25^ The use of bioorthogonal sugars has helped to increase our understanding of glycosylations.^26–29^ Functional group modifications around the sugar hydroxyl groups are tolerated by the biosynthetic pathways and transform them into the corresponding sugar-nucleotide donor. Glycosyltransferases can utilize these unnatural monosaccharide donors for glycosylation of proteins, for example, OGT using a sugar donor containing the reactive azide group (UDP-GlcNAz) donor to deposit an *O*-GlcNAz modification onto intracellular proteins.^26^ Recently, bioorthogonal sugars have been employed to exploit salvage pathways to see if their permissive nature extended to smaller chemically modified groups along the 2-acetamido group of GlcNAc on MCRs. These MCRs have proven widely useful for assessing *O*-GlcNAcylated proteins,^30^ mapping *O*-GlcNAc to the genome,^31^ imaging *O*-GlcNAcylated proteins in situ,^32^ and uncovering fundamental biology^33, 34^. However, no MCR has been developed to specifically incorporate into *N*-linked glycans. Design and synthesis of selective MCRs for *N*-linked glycans have proven difficult due to the overlap of common monosaccharide building blocks in the glycan assembly and inefficient turn-over by salvage pathway enzyme.^35^ Only one successful example comes from the incorporation of GlcNAlk and GlcNAz onto glycans in yeast.^36^ Thus, the use of bioorthogonal chemistry to study glycosylation has proven to be an integral tool for the field, allowing discovery of new classes of glycans, like glycosylated small RNAs.^16^

Continuing previous efforts using bioorthogonal sugars^21, 37–40^ and especially *N*-glycans,^41^ we now report synthesis of a next generation MCR for the specific labeling of hybrid *N*-glycan structures. We used a series of *N*-glycan trimming pathway inhibitory experiments along with MGAT1 siRNA to demonstrate that this MCR is specific and enzymatically incorporated into hybrid N-linked structures. Surprisingly, we find significant hybrid *N*-glycans in the nucleus. We identify the nucleolar protein fibrillarin as being modified with hybrid *N*-glycan structures. To our knowledge, this is the first MCR specifically labeling *N*-glycan hybrid structures. This MCR should allow a more detailed analysis of the biogenesis and intracellular fate of hybrid N-linked glycans.

## Results.

### Design, synthesis, and incorporation of 1,3-Pr_2_-6-OTs GlcNAlk (MM-JH-1, 6)

**Figure 1.**
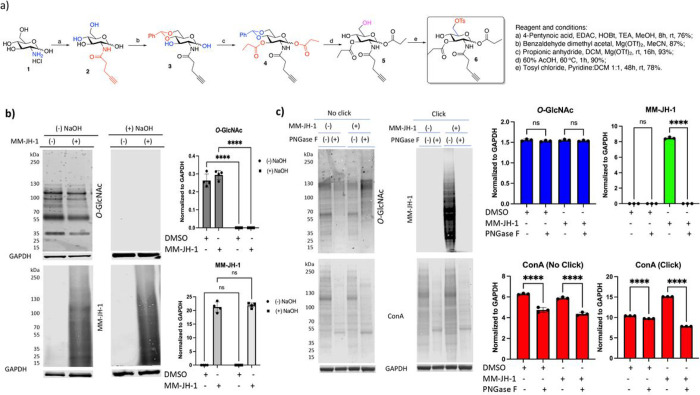
Compound MM-JH-1 was specific to N-linked glycosylation (A) Chemical synthesis of the title compound MM-JH-1. (b) β-elimination study showing the labeling are not O-linked, with MM-JH-1 (detected with RL2 antibody) signal remaining after incubation with NaOH. Assessment of O-GlcNAc (detected with RL2 antibody) was used as a control to ensure successful β-elimination (N = 4; An ordinary one-way ANOVA test shows ****p < 0.0001, ns = not significant). (c) PNGase F treatment removed the MM-JH-1 labeled signals. RL2 signal remained unchanged while ConA signal decreased, indicating PNGase F properly removed N-Linked glycans. Graph showing quantifications are to the right of the respective blots (N = 3; An ordinary one-way ANOVA test shows ****p < 0.0001, ns = not significant, error bar represents standard deviation).

Bioorthogonal sugars, including those labeled by azides and alkynes, have shown utility for identifying glycosylated proteins in both tissue culture cells and whole organisms.^21, 42–46^ While MCRs have great potential as tools for studying glycans, it has been reported that all commonly used MCRs undergo non-enzymatic *S*-glycosylation. Because of the off-target incorporations, these unnatural monosaccharides are not ideal for specific metabolic glycan labeling.^47, 48^ Recently,1,3-di-*O*-acylated GalNAz (1,3-Ac_2_GalNAz and 1,3-Pr_2_GalNAz) compounds were reported to be relatively more specific towards glycan labeling with oligomembrane permeability. Importantly, the 1- and 3-hydroxyl group protected with longer alkyl chain, i.e. a propionyl group, showed better membrane permeability and lower acyl migration tendency,^49^ compared to its acetate protected analogue.^48^ *p*-toluene sulfonyl (Tosyl) group had previously been used for ligand directed metabolic labeling of proteins,^50^ and specifically 6-OTs derivative of d-galactose was described as chemically analogous to the enzyme bound intermediate towards formation of the corresponding 6-phosphate.^51^ Thus, we chemically synthesized 2-deoxy-2-*N*-pentynoylamide-1,3-di-*O*-propionyl-6-*O*-tosyl-d-glucopyranoside (1,3-Pr_2_-6-OTsGlcNAlk, **6**). GlcNAlk (**2**) was prepared as previously reported^46^ starting from d-glucosamine hydrochloride. GlcNAlk (**2**) was converted to 1,3-Pr_2_-6-OTs GlcNAlk (MM-JH-1, **6**) through one-pot sequential functional group modifications (Figure 1a, for detailed experimental procedures see materials and methods section) resulting with the final product in 56.8% overall yield. HRMS analysis ([M+Na]^+^ calculated for C_24_H_31_O_10_NSNa 548.1566 and found 548.1567; Mass of oxocarbenium ion calculated for C_21_H_26_O_8_NS^+^ 452.1 and found 452.2) along with detailed NMR spectral analysis where peaks for two (2) doublet protons (4 protons total) in aromatic region (7–8 ppm) and -CH3 peak at 21.6 ppm in ^13^C NMR showing *p*-Me substituted aromatic ring and correlation of hydroxyl proton signal at 3.04 ppm with *H*-4 signal unambiguously confirmed the formation of 4-hydroxyl group free 6-OTs protected GlcNAlk derivative **6** (Supplemental Fig. 1, 2 and 3).

With this compound in hand, we next evaluated cytotoxicity and the extent of incorporation inside cells. Cytotoxicity of the GlcNAlk compound (MM-JH-1) was evaluated by treating HeLa and mouse embryonic fibroblast (MEF) cells with varied concentrations for 48 h (Supplemental Fig. 4 and 5). To assess incorporation, we used on lysates from MM-JH-1 treated cells to TAMRA-label modified proteins and detect labelled proteins using an anti-TAMRA antibody. Increasing concentrations of MM-JH-1 resulted in significantly higher incorporation in a concentration-dependent manner in the range of 25–500 μM. No significant cytotoxicity was observed at concentrations up to 200 μM, while some minimal cytotoxicity was observed at 500 μM (Supplemental Fig. 4 and 5). With significant amount of metabolic incorporation and no significant cytotoxicity, 100 μM concentration of the compound MM-JH-1 was chosen for further studies.

Next, we set out to determine the linkage by which this bioorthogonal sugar was incorporated. β-Elimination is a technique used to cleave *O*-linked glycans while leaving *N*-linked glycans unharmed.^43, 52^ *O*-Linked glycans are sensitive to β-elimination and it would be expected that *O*-linked MM-JH-1 would also be base-labile. To assess if MM-JH-1 was incorporated through an *O*-linkage, we used click chemistry on lysates from MM-JH-1 and DMSO control cells to TAMRA-label modified proteins. After the click reaction, lysates were run on an SDS gel and transferred to a nitrocellulose membrane. The membrane was then incubated overnight in 55 mM NaOH or water. After the β-elimination, we used a TAMRA antibody to detect incorporation of MM-JH-1. The β-elimination condition did not show any loss in signal for TAMRA-labeled proteins coming from the labeling by MM-JH-1 (Figure 1b), but under the same condition the signals for the intracellular *O*-GlcNAc modified proteins (detected with RL2 antibody) were eliminated on the blot (Figure 1b). These data suggest that the alkyne-derived signal was not due to alkyne-modified *O*-linked glycans.

To determine whether signal from the alkynyl sugar MM-JH-1 was due to *N*-linked glycans, the cell lysates were treated with PNGase F, a well-characterized glycosidase for enzymatic cleavage of *N*-linked glycans.^44, 52, 53^ We observed complete loss of TAMRA-labeled signal upon immunoblotting, suggesting that the signal was due to labeling of *N*-linked glycans (Figure 1c). Loss of total lectin binding signals (determined by Concanavalin A (ConA) blotting, Figure 1c) for both clicked and non-clicked cell lysates further validated PNGase F treatment for detection of *N*-linked glycans.

### Tunicamycin inhibits incorporation of MM-JH-1

**Figure 2.**
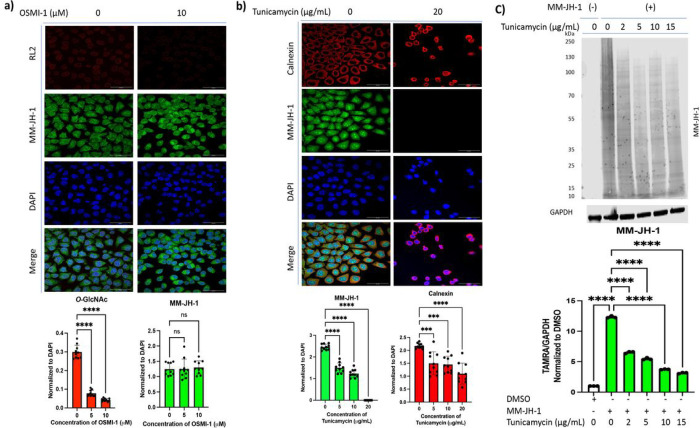
The compound MM-JH-1 was enzymatically added to the N-linked glycans. (a) Cells were treated with or without MM-JH-1 and with or without OGT inhibitor OSMI-1 as indicated. Whereas O-GlcNAc staining (red) diminished, there was no effect on labeling by this compound (green, AF 488). Quantification of images were done by normalizing mean fluorescent signal to DAPI (blue) are shown to the right (b) Cells were treated or left untreated with Tunicamycin, an inhibitor of N-linked glycosylation, as indicated. Treatment diminished labeling by MM-JH-1 (green, AF 488) shown by confocal imaging. Calnexin (red) was used to assess successful tunicamycin treatment. Quantification of images were done by normalizing mean fluorescent signal to DAPI (blue) are shown to the right. Quantification of images were done by normalizing mean fluorescent signal to DAPI (blue) are shown under the images. (N = 3; n = 10; An ordinary one-way ANOVA test shows ****p < 0.0001, ***p = 0.0001; N = Number of experiment repeat, n = Number of individual cells chosen for quantification of the confocal images, scale bar = 50 mm). (c) Increasing amounts of Tunicamycin (as indicated) inhibited labeling by MM-JH-1 shown by western blotting. (N = 3; An ordinary one-way ANOVA test shows ****p < 0.0001, ns = not significant, error bar represents standard deviation).

Non-enzymatic glycosylation using MCRs is a well-described artifact of using unnatural sugars.^47^ To determine that the alkynyl sugar MM-JH-1 is enzymatically incorporated, and to corroborate our β-elimination and PNGase F studies, we used a series of glycosyltransferase inhibitors in cells treated with MM-JH-1 or DMSO. First, we used the *O*-GlcNAc transferase (OGT) inhibitor OSMI-1^54^ and determined incorporation using fluorescence confocal microscopy, which would allow us to determine levels and localization changes after inhibition. Here, a click reaction was used to add an Alexa Fluor 488 to the MM-JH-1 sugar. With increasing OSMI-1 concentration, we observed no change in the Alexa Fluor 488 signal (MM-JH-1 sugar), despite significant loss of endogenous *O*-GlcNAc (RL2) signal on confocal imaging (Figure 2a and Supplemental Fig. 6). This indicated that labeling by MM-JH-1 was independent of OGT, and hence it was not incorporated into *O*-GlcNAc.

Next, we assessed the impact of Tunicamycin, an inhibitor of *N*-linked glycosylation.^55^ With increasing tunicamycin concentration, loss of signal from the compound MM-JH-1 was observed on both confocal imaging (Figure 2b, Supplemental Fig. 7) and western blotting (Figure 2c). To confirm Tunicamycin treatment was inhibiting *N*-glycan synthesis we used Calnexin staining to detect significant ER stress (Figure 2b). Together, these data suggested that labeling by our alkynyl sugar MM-JH-1 was enzymatic and specifically incorporating into *N*-linked glycosylation.

### MM-JH-1 labels hybrid structures of *N*-glycans

**Figure 3.**
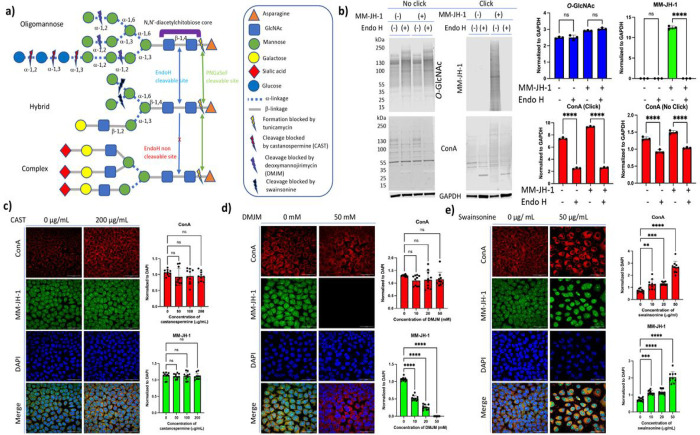
The compound MM-JH-1 incorporates into hybrid structures of N-glycans. (a) Picture indicating types of N-glycan structures and where inhibitors act. (b) Endo H treatment of immunoblots removed the labeling by the compound MM-JH-1, as indicated by TAMRA. Assessment of O-GlcNAc and ConA were used as controls to ensure efficacy of Endo H reaction with quantification shown to the right of blots. (c) Glucosidase inhibitor Castanospermine (CAST) had no effect on the labeling by MM-JH-1 (green). (d) Mannosidase-I inhibitor 1-Deoxymannojirimycine (DMJM) reduced the signals of labeling by MM-JH-1 (green). (e) Mannosidase-II inhibitor Swainsonine increased the signals of labeling by MM-JH-1 (green). All quantification of images were done by normalizing mean fluorescent signal to DAPI (blue) are shown to the right of the respective images. (N = 3; n = 10; An ordinary one-way ANOVA test shows ****p < 0.0001, ***p = 0.0001, ns = not significant, scale bar = 50 mm, error bar represents standard deviation).

Next, we used a series of enzymes and inhibitors to determine the class of *N*-linked glycan structures in which MM-JH-1 is being incorporated. Endo H is an enzyme that hydrolyses the bond between the two GlcNAc units that comprise the chitobiose core of the oligomannose and hybrid type *N*-glycan structures but not complex type glycans (Figure 3a).^56^ Endo H treatment resulted in complete removal of the TAMRA-labeled signal and significant loss of ConA signal on western blotting (Figure 3b). These data suggest that the alkynyl sugar MM-JH-1 is not incorporated into complex type *N*-glycan structures.

Glucosidase-I and II are the two enzymes that cleave the three glucose residues consecutively in the oligomannose type glycans and starts the *N*-glycan trimming process.^17^ The removal of these glucose residues can be prevented by the use of glucosidase inhibitors like castanospermine (CAST)^55^ and 1-deoxynojirimycin (DNJM),^55^ and in such cases *N*-glycans retain the three glucose residues and lose one or two mannose residues proceeding to the further trimming of the oligosaccharide.^17^ HeLa cells at 50–60% confluency were treated with different concentrations of CAST (50–200 μg/mL)^55^ or DNJM (50–200 mM)^55^ for 12 h followed by additional 48 h incubation with 100 μM of MM-JH-1, cells were fixed, permeabilized and subjected to click reaction with AF488 and incubated with ConA and DAPI prior to immunofluorescence imaging. Using these inhibitors, we observed no change in either MM-JH-1 labeled signal (detected with AF488) or total lectin binding signal (detected with ConA) (Figure 3c, Supplemental Fig. 8 and 9), further suggesting that the alkynyl sugar MM-JH-1 was not incorporated into the complex type *N*-Glycan structures.

Mannosidase-I is an ER resident enzyme that cleaves the Man-α(1–2) linkages in partially trimmed *N*-glycans producing Man_5_GlcNAc_2_Asp unit, the base structure of hybrid type *N*-glycans.^17^ With increasing concentration of mannosidase-I inhibitors such as 1-deoxymannojirimycin (DMJM, 5–50 mM)^55^ or kifunensine (1–5 mM)^55^ signal intensity for MM-JH-1 labeled signal detected with both AF488 (Figure 3d, Supplemental Fig. 10 and 11) and Oregon Green 488 (Supplemental Fig. 12) decreased while there was no change in ConA signal. These data indicate that the signal for alkynyl sugar MM-JH-1 was independent of the fluorophore used for detection and that the label was present on the side chain of the hybrid structures.

Mannosidase-II is a Golgi resident enzyme that breaks all the terminal Manα1–3 and Manα1–6 linkages at hybrid type glycans processing them further to complex *N*-glycan structures.^17^ Swainsonine, a very well-known mannosidase-II inhibitor^57^ blocks the conversion of hybrid type *N*-glycan to the complex structures and thus increasing the total amount of hybrid *N*-glycan structures.^58^ When this inhibitor was used on MM-JH-1 or DMSO treated cells, we observed a significant increase in MM-JH-1 labeled signal with increasing concentration of swainsonine (10–50 μg/mL, Figure 3e, Supplemental Fig. 13).

To confirm the presence of MM-JH-1 in hybrid N-linked structures, we performed MALDI-MS analysis of the oligosaccharides released by Endo H digested MM-JH-1 treated cells. This analysis shows the presence of a peak differing from the endogenous by 248 Da. This mass difference of 248 Da can be attributed to the replacement of one hexose unit carrying 204 Da with MM-JH-1 GlcNAlk derivative with mass contribution of 452 Da, and compound MM-JH-1 has a signature mass of 452 Da for its corresponding oxocarbenium ion (Supplemental fig. 14). Therefore, our data thus far was consistent with the alkynyl sugar MM-JH-1 being incorporated into the hybrid class of *N*-glycan structures.

### MM-JH-1 is enzymatically incorporated into hybrid structures by MGAT1

**Figure 4.**
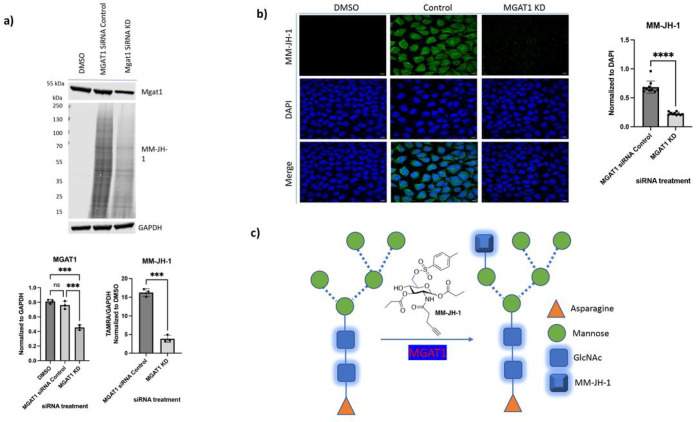
The compound MM-JH-1 was added to glycans by MGAT1. (a) siRNA against MGAT1 reduced MGAT1 levels by about 50%. MGAT1 knockdown reduced the signal of labeling by MM-JH-1 on western blotting as detected by TAMRA. Quantification is shown below blots (N = 3; For MGAT1 an ordinary one-way ANOVA test shows ****p < 0.0001; ***p = 0.0003, ns = not significant and for TAMRA an unpaired t test shows ***p = 0.0001). (b) MGAT1 knockdown reduced the signal of labeling by MM-JH-1 (green) on confocal imaging. Quantification is shown to the right of images (N= 3; n =10; An an unpaired t-test shows ****p < 0.0001; ***p = 0.0003, ns = not significant, scale bar = 10 mm, error bar represents standard deviation). (c) Schematic representation of addition of MM-JH-1 by MGAT1 to the core of oligo mannose structures and the resulting hybrid structures.

Our inhibitor and mass spectrometry studies were consistent with the alkynyl sugar MM-JH-1 being specifically incorporated into hybrid structures, thus, we decided to focus on MGAT1. MGAT1 is the *N*-acetylglucosaminyltransferase (GlcNAcT-I) that initiates the formation of hybrid and complex *N*-glycans by addition of a GlcNAc-β(1–2) residue to the core Man-α(1–3) residue. In the absence of MGAT1, *N*-glycans of mature glycoproteins are solely composed of oligomannose structures.^59^ To determine if the incorporation of MM-JH-1 required MGAT1, we knocked down MGAT1 by siRNA. Using this technique, we were able to decrease MGAT1 levels by about 50% (Figure 4a) and significantly decrease MM-JH-1 incorporation as measured by immunoblot using TAMRA (Figure 4a) and confocal imaging (Figure 4b). Taken together, these data are consistent with the proposal that MM-JH-1 is enzymatically incorporated into the side chain of *N*-glycan hybrid structures by the activity of MGAT1 (Figure 4c).

### MM-JH-1 labeled signal colocalizes with the nucleolus

Throughout our experiments, we have consistently noticed MM-JH-1 labelling within the nucleus. Based on the inhibitor and MGAT1 knockdown studies, this labelling is both specific and enzymatic. This observation was quite surprising as nuclear *N*-linked glycosylation has not been widely observed. To confirm this observation, we fractionated nuclear and cytosolic lysates from MM-JH-1 and DMSO treated cells, ran the lysates out on a gel, blotted and performed PNGase F and Endo H assays as described earlier. As we had observed in whole cell lysates (Figures 1C and 3B), the TAMRA signal was readily detectable in the nuclear lysates and disappeared when these enzymes were used (Supplemental fig.15 and 16). Based on confocal imaging, the MM-JH-1 incorporation was present in the nucleolus. Therefore, we focused our efforts on investigating nucleolar proteins as candidates for attachment of the hybrid N-glycans.

**Figure 5.**
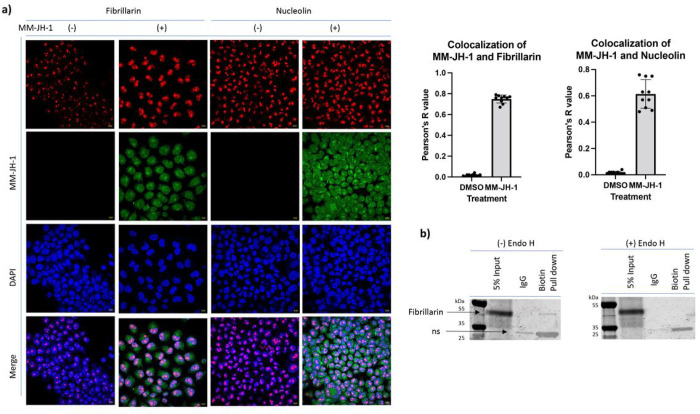
The nucleolar protein Fibrillarin is modified by the MM-JH-1 compound. (a) Colocalization of fibrillarin and nucleolin signal (red) with MM-JH-1 (green). Colocalization was determined by Pearson’s R value as indicated to the right of images. (b) After performing a click reaction with a biotin-alkyne, Streptavidin was used on lysates to pulldown MM-JH-1 labelled proteins. 5% of input, flowthrough from an IgG elution buffer (to assess non-specific interactions), and the Biotin/Streptavidin pulldown were all run out on a gel, transferred to a membrane and immunoblotted using a fibrillarin antibody (left panel). A band indicating fibrillarin is visible in the 5% input and biotin-pull down lanes. These lysates were treated with Endo H, run out on a gel and immunoblotted for fibrillarin. In this case fibrillarin signal is lost in the biotin pull down lane indicating that Endo H cleaved the sugar from fibrillarin. (N= 3; n =10, scale bar = 10 mm)

First, we assessed co-localization with two nucleolar proteins, nucleolin and fibrillarin, both of which have been reported to potentially contain N-linked glycosylations.^60–62^ Here, we found significant colocalization of MM-JH-1 labeled signals with both nuclear markers (detected with anti-fibrillarin nucleolar marker antibody, and anti-nucleolin antibody) suggesting MM-JH-1 was labeling proteins within the nucleolus (Figure 5a). To further confirm co-localization, actinomycin D was used to disrupt the organization of the nucleolus.^63^ We again assessed co-localization of the MM-JH-1 signal with fibrillarin and nucleolin. We found that after actinomycin D treatment, fibrillarin maintained localization with a Pearson’s R value of about 0.6 (Supplemental Fig. 17), whereas for nucleolin the Pearson’s R value decreased to about 0.2 (Supplemental Fig. 18). Thus, the nucleolar signal of the MM-JH-1 closely mimicked that of the fibrillarin signal. To further assess fibrillarin as modified by our compound, we treated cells with MM-JH-1 or DMSO, collected cellular extracts and used Click chemistry to add an acid cleavable biotin onto the labelled proteins. A Streptavidin-conjugated bead was used to enrich for biotin containing proteins. After washing, the samples were incubated in neutral pH IgG buffer as a control for non-specific binding to the beads. Enriched proteins were then boiled off the bead, run out on a gel, and immunoblotted using an antibody against fibrillarin. We detected fibrillarin in the biotin-enriched samples (Figure 5b). Importantly, when treated the lysate with Endo H, fibrillarin could no longer be detected, confirming modification of fibrillarin. Thus, using this MM-JH-1, we were able to uncover hybrid *N*-Linked glycosylation on the nucleolar protein fibrillarin.

## Discussion.

Clickable unnatural monosaccharides have proven to be powerful tools towards metabolic glycan engineering (MGE) for the introduction of unnatural functional groups into cellular glycans and profiling of cellular glycosylations.^64^ While the use of MCRs have been important in progressing the field of glycobiology, recent studies have uncovered non-enzymatic artificial *S*-glycosylation with the commonly used MCRs meant to incorporate into intracellular *O*-GlcNAcylation. The reaction between MCRs with free thiol of cystine residues calls into question the conclusions garnered from using these MCRs towards detection of *O*-GlcNAc.^47, 48^ This is particularly significant for MCRs targeting the intracellular *O*-GlcNAcylation due to high residence of free cystine sulfhydryl groups compared to the cell surface, where many cystine residues are found as oxidized disulfides.^52^ In the search for better MCRs, 1,3-Pr_2_ GalNAz was introduced as a next generation unnatural monosaccharide that can avoid artificial *S*-glycosylation reaction.^48^ In this study we have modified the 6-hydroxyl group of 1,3-Pr_2_ GlcNAlk with 6-*O*-Tosyl and looked for its specificity as an MCR. Treatment of HeLa cells with this compound was not significantly toxic up to 500 μM concentration. NaOH treatment (β-elimination condition) or *O*-GlcNAc inhibition (OSMI-1) had no effect on labeling by this compound. However, PNGase F treatment and *N*-glycan synthesis inhibitor (tunicamycin) blocked labeling by this alkyne sugar, suggesting that this MCR was enzymatically incorporated into the *N*-glycan structures. Using a series of specific inhibitors and MALDI-MS glycan analysis, we determined MM-JH-1 was likely incorporated into hybrid structures. Further, we confirmed this by knocking down MGAT1 and detecting a concomitant reduction in MM-JH-1 incorporation. Thus, this data indicates that MM-JH-1 specifically and enzymatically labelled hybrid structures of *N*-linked glycans.

The enzymatic steps required for the formation of complex *N*-glycan structures follows a discrete hierarchy depending on substrate specificities of the transferases. The first step in the glycan branching process is the addition of a GlcNAc-β(1–2) residue transferred from UDP-GlcNAc by MGAT1, forming the GlcNAcMan_5_GlcNAc_2_-Asp core hybrid structure. This addition by MGAT1 is required for several subsequent modifications. The GlcNAc-Man_2_ residue in the core hybrid structure acts as a ‘recognition arm’ for other enzymes like Golgi α-Mannosidase-II, MGAT2, MGAT3, MGAT4, MGAT5 and FUT8, but not B4GALT1 (for addition of a β(1–4)Gal residue).^65^ Those MGAT1 dependent enzymes bind the recognition arm using a broad exosite surface that extends away from the active site and forms a specific pocket for the terminal GlcNAc residue. In consideration of how the MM-JH-1 might be utilized, the *N*-acetyl group of UDP-GlcNAc is deeply buried inside the binding pocket of MGAT1 structure,^66^ leaving enough space to accommodate the bulky 6-*O*-Tosyl functionality of our compound and allow transfer by MGAT1. On the other hand, the terminal GlcNAc structure is essential for its binding in the exosite pocket of downstream enzymes and our modified GlcNAlk derivative MM-JH-1 might not being able to fit in the site, inhibiting further *N*-glycan processing. Thus, rendering incorporation of MM-JH-1 specific to hybrid structures.

Surprisingly, we found significant colocalization of MM-JH-1 with the nucleolus. Colocalization and immunoprecipitation of fibrillarin with MM-JH-1 suggests that this nucleolar protein is *N*-linked glycosylated (Figure 5a and 5b). Fibrillarin has a relatively small molecular weight (37 kDa) and can move in and out of the nuclear membrane.^60^ Importantly, it has a potential *N*-glycosylation site at Asparagine 80 (NetNGlyc-1.0). While our study uncovered fibrillarin as *N*-linked glycosylated, it is likely not the only nuclear protein modified. Further research is necessary to determine the extent of nuclear *N*-linked glycosylation, the function of this modification in the nucleus, and how these proteins encounter MGAT1.

Use of specific *N*-linked incorporated bioorthogonal sugars have great potential as tools in biomedical research. Changes in *N*-glycan structures are considered to be important for the epithelial–mesenchymal transition (EMT) and the resultant change of adhesive properties of cancer cells.^67^ Aberrant glycosylation has been pinpointed as a hallmark of cancer due to its contributions in carcinogenesis, cancer progression and metastasis, conferring a new perspective for cancer research involving underlying mechanisms investigation and clinical translation and application.^68^ Most of the cancer biomarkers that are in use today are glycoproteins or glycolipids, and they are measured immunochemically using monoclonal antibodies. The epitope for these monoclonal antibodies against glycoproteins are mostly toward the protein moiety and not toward the glycan structures.^67^ Currently, however, it is difficult to detect the early stage of cancer by using these antibodies. Thus, bioorthogonal sugars have the potential to detect early-stage changes in *N*-glycan structures that can lead to the earlier detection of cancer. Additionally, a better understanding and specific labelling of hybrid *N*-glycans has great potential. Hybrid *N*-glycans play an important role as ligands for HIV-1 neutralizing antibodies including PG9, PG16, VRG26.25 and VRC26.09, suggesting that such glycans can serve as epitopes for HIV vaccine.^69^ In addition, hybrid *N*-glycans modify protein properties such as binding of IgG_1_ to Fcγ receptor IIIa and cell-cell interaction mediated by E-cadherin.^19, 70^ To our best knowledge, MM-JH-1 is the first MCR that specifically labels only the *N*-glycan hybrid structures. Now, using this MCR, hybrid *N*-glycans can be distinguished clearly from the complex type, and thus one can use this tool to undertake more specific in-depth studies of this ‘intermediate’ type of *N*-glycan.

## Materials and methods.

### Reagents

All chemicals, reagents, and general lab supplies were purchased from Thermo Fisher Scientific or Sigma-Aldrich unless noted. GlcNAlk was prepared as previously reported.^46, 71^ Cell culture reagents including DMEM with 2 mM glutamax was purchased from Gibco. Alexa Fluor 488 azide (Thermo Fisher Scientific A10266), Oregon Green 488 azide (Thermo Fisher Scientific A10180), TAMRA azide (Thermo Fisher Scientific T10182), and Biotin DADPS picolyl azide (Sussex Research, BL000010) were dissolved at a stock concentration to 1 mM in DMSO.

Primary antibodies used include those for the following epitopes (catalog number): Mouse anti *O*-GlcNAc (RL2) (Thermo Fisher Scientific MA1-072), Mouse anti GAPDH (Abcam ab8245), Mouse anti MGAT1 (Abcam, ab 180578), Rabbit anti TAMRA (Thermo Fisher Scientific, A6397), Rabbit anti GAPDH (Abcam, ab18078), Rabbit anti nucleolin (Sigma, N2662), Rabbit anti Fibrillarin (Abcam, ab5821), Rabbit anti Calnexin (Abcam, ab 22595), Mouse anti Histone H3 (Cell Signaling Technology, 3638), Rabbit anti Histone H3 (Cell Signaling Technology, 3717), Concanavalin A Texas Red (ConA, Thermo Fisher Scientific C825), Biotin conjugated ConA (Vector Lab, B-1005-5).

Inhibitors were purchased from Sigma Aldrich with the following specifications. OSMI-1 (Sigma-Aldrich, SML1621), Tunicamycin (Sigma-Aldrich, T7765), Castanospermine (Sigma-Aldrich, 218775), 1-Deoxynojirimycin (DNJM, Sigma-Aldrich, 08012), 1-Deoxymannojirimycin (DMJM, Sigma-Aldrich, D9160), Swainsonine (Sigma-Aldrich, S9263), Kifunensine (Sigma-Aldrich, 422500), Actinomycin D (Sigma-Aldrich, A9415).

### General methods for chemical synthesis:

Unless specified otherwise, all reagents and solvents were purchased from Thermo Fisher Scientific or Sigma-Aldrich Chemical Company and used as supplied. Reactions were monitored by thin-layer chromatography (TLC) on silica gel 60 glass slides. Spots were visualized by charring with H_2_SO_4_ in EtOH (5% v/v) and/or UV light. Solutions in organic solvents were dried with anhydrous MgSO_4_ and concentrated at reduced pressure at < 40 °C.

### Chemical analysis instruments:

NMR spectra were measured at 25 °C for solutions in CDCl_3_, at 500 MHz or 600 MHz for 1H, and at 125 MHz or 150 MHz for ^13^C with Bruker Avance Spectrometers. Assignments of NMR signals were aided by 1D and 2D experiments (^1^H–^1^H homonuclear decoupling, APT, COSY, HSQC, TOCSY and HMBC) run with the software supplied with the spectrometer. Chemical shifts were referenced to that of tetramethylsilane (0 ppm) or signals of residual non deuterated solvent (7.16 for ^1^H) and for ^13^C, signal of the solvent (CHCl_3_, 77.00 ppm). Topshim (v12.0.3) was used for all chemical NMR analysis. Data are reported as chemical shift, multiplicity (brs, broad signal; s, singlet; d, doublet; t, triplet; q, quartet; quin, quintet; sept, septet; m, multiplet), coupling constants in Hertz (Hz) and integration. High-resolution mass spectrometric (HRMS) measurements were performed on a proteomics optimized Q-TOF-2 (Micromass-Waters) using external calibration with polyalanine, unless otherwise noted. Observed mass accuracies are those expected based on known instrument performance as well as trends in masses of standard compounds observed at intervals during the series of measurements. Reported masses are observed masses uncorrected for this time-dependent drift in mass accuracy.

### Synthesis:

2-deoxy-2-*N*-pentynoylamide-1,3-di-*O*-propionyl-6-*O*-tosyl-d-glucopyranoside (1,3-Pr_2_-6-OTs-GlcNAlk, 6):

4-Pentynoic acid (600 mg, 6.1 mmol) was added to a stirring solution of d-glucosamine hydrochloride (875 mg, 4.06 mmol) and TEA (2 mL) in methanol (20 mL) at room temperature. The mixture was stirred under room temperature for 30 min, then moved into ice bath, HOBt (550 mg, 4.06 mmol) and EDC hydrochloride (780 mg, 4.06 mmol) was added. Then the reaction was stirred at room temperature for 8 h, when TLC (DCM:MeOH 4:1, R_*f*_ = 0.48) showed complete consumption of starting material with a couple of newly formed much faster moving spots. The mixture was concentrated and briefly purified by column chromatography (silica gel; DCM:MeOH = 8:1) to give 2-deoxy-2-*N*-pentynoylamide-d-glucopyranose (**2**) as white solid (801 mg, 76%). Thus, purified product was characterized with HRMS (HRMS (ESI-TOF): *m/z* [M+Na^+^] calcd for C_11_H_17_NO_6_Na 282.0954; found 282.0958) and proceed further to the next step reaction. 0.6 mL of benzaldehyde dimethyl acetal was added to the stirring solution of **2** (700 mg, 2.7 mmol) in dry acetonitrile (MeCN, 50 mL) followed by addition of magnesium trifluoromethanesulfonate (Mg(OTf)_2_,^72^ 87 mg, 0.27 mmol) and left on stirring at room temperature for the weekend. After 2 days, TLC (DCM:MeOH 9:1, R_*f*_ = 0.54) shows faster moving spot were formed. The reaction was neutralized with NEt3, solvent was evaporated under vacuum and the formation of the desired product **3** (815 mg, 87%) was confirmed by HRMS analysis (HRMS (ESI-TOF): *m/z* [M+H^+^] calcd for C_18_H_22_NO_6_ 348.1447; found 348.1446). This crude mixture was dried under vacuum and subjected to further acetylation reaction.

Propionic anhydride (0.9 mL, 6.9 mmol) and Mg(OTf)_2_ (37 mg, 0.12 mmol) was added to the stirring solution of **3** (800 mg, 2.3 mmol) in dry DCM (10 mL) and left stirring for overnight. After 16 h, TLC (9:1 DCM:MeOH, R_*f*_ = 0.62) then showed consumption of the starting material and formation of the product which appeared as faster moving and two poorly separated spots representing a mixture of anomers. The reaction mixture was quenched with MeOH and evaporated under vacuum. The crude mixture was dissolved in DCM and subsequently washed with 1(N) HCl, saturated NaHCO3 and brine solution. The combined washings were collected, dried over Na_2_SO_4_. The crude product (**4**, 984 mg, 93%, HRMS (ESI-TOF): *m/z* [M+Na^+^] calcd for C_24_H_29_NO_8_Na 482.1791; found 482.1789) was collected as colorless syrup and proceeded to the next step reaction. 10 mL of 60% Acetic acid^73^ was added to the **4** (950 mg, 2.1 mmol) and left on stirring for 1 h at 60 °C. TLC (3:1 EA:Hexane, R_*f*_ = 0.27) then showed consumption of the starting material and formation of a much slower moving spot. Acetic acid was removed under vacuum and the crude mixture was purified by column chromatography (3:1 EA:Hexane) to give pure product (**5**, 690 mg, 90%) as colorless syrup. ^1^H NMR for the major β anomer (600 MHz, CDCl3): δ 6.17 (d, 1H, *J* = 3.7 Hz, *H*-1), 6.09 (d, 1H, *J* = 8.9 Hz, -N*H*), 5.18 (dd, 1H, *J* = 9.5 Hz, 10.8 Hz, *H*-3), 4.38 (m, 1H, *H*-2), 3.94 (m, 1H, *H*-4), 3.91–3.80 (m, 2H, *H*-6,6’), 3.72 (m, 1H, *H*-5), 3.11 (brs, 1H, -O*H*), 2.49–2.31 (m, 8H, 4 ×C*H*_2_), 2.00 (1H, brs, -O*H*), 1.98 (1H, t, *J* = 1.98 Hz, -C*H*), 1.81 (3H, t, *J* = 7.6 Hz, C*H*_3_), 1.14 (3H, t, *J* = 7.5 Hz, C*H*_3_); ^13^C NMR (150 MHz, CDCl_3_): δ 175.9 (*C*=O), 172.9 (*C*=O), 171.3 (*C*=O), 90.7 (*C*-1, *J*_C-1,H-1_ = 177.8 Hz), 73.5 (*C*-5), 72.8 (*C*-3), 69.5 (-*C*H), 67.4 (*C*-4), 61.0 (*C*-6), 51.1 (*C*-2), 34.9 (*C*H_2_), 27.5 (*C*H_2_), 27.4 (*C*H_2_), 14.6 (*C*H_2_), 9.1(*C*H_3_), 8.8 (*C*H_3_); HRMS (ESI-TOF): *m/z* [M+Na] calcd. for C_17_H_25_NO_8_Na 394.1478; found 394.1474.

Tosyl chloride (280 mg, 1.48 mmol) was added to the stirring solution of **5** (500 mg, 1.35 mmol) in 1:1 mixture of dry pyridine and dry DCM (10 mL) at 0 °C and left on stirring at room temperature. After 48 h when TLC (1:1 EA:Hexane, R_*f*_ = 0.30) showed consumption of the starting material and formation of a faster moving spots, the reaction mixture was quenched with MeOH and evaporated under vacuum. The crude mixture was dissolved in DCM and subsequently washed brine solution. The combined washings were collected, dried over Na_2_SO_4_ and evaporated under vacuum to give crude reaction mixture as light-yellow syrup. Column chromatographic purification of this crude mixture (1.2:1 Hexane:EA) gave pure product (**6**, 552 mg, 78%) as white foam. ^1^H NMR for the major β anomer (500 MHz, CDCl_3_): δ 7.82–7.75 (d, 2H, *J =* 8.5 Hz, Ar*H*), 7.38–7.33 (d, 2H, *J =* 8.2 Hz, Ar*H*), 6.07 (d, 1H, *J* = 3.7 Hz, *H*-1), 5.84 (m, 1H, -N*H*), 5.13 (dd, 1H, *J* = 10.9 Hz, 8.8 Hz, *H*-3), 4.43 (dd, 1H, *J* = 9.1 Hz, 2.5 Hz, *H*-6), 4.31 (m, 1H, *H*-2), 4.18 (dd, 1H, *J* = 1.4 Hz, 10.9 Hz, *H*-6’), 3.89–3.81 (m, 2H, *H*-4, *H*-5), 3.04 (m, 1H, -O*H*), 2.48–2.27 (m, 11H, 4 ×C*H*_2_, *p*-C*H*_3_ at 2.46 ppm), 1.95 (t, 1H, *J* = 2.6 Hz, -C*H*), 1.79–1.12 (2t, 6H, *J* = 7.3 Hz and 7.0 Hz, 2×C*H*_3_); ^13^C NMR (125 MHz, CDCl_3_): δ 175.8 (*C*=O), 172.3 (*C*=O), 170.9 (*C*=O), 145.3, 132.4, 129.9 (2*C*), 127.9 (2*C*), 90.4 (*C*-1), 72.5 (*C*-3), 71.7 (*C*-5), 69.5 (-*C*H), 67.7 (*C*-6), 67.4 (*C*-4), 51.0 (*C*-2), 35.1 (*C*H_2_), 27.6 (*C*H_2_), 27.4 (*C*H_2_), 21.6 (*C*H_3_), 14.6 (*C*H_2_), 9.0 (*C*H3), 8.8 (*C*H_3_); HRMS (ESI-TOF): *m/z* [M+Na] calcd. for C_24_H_31_O_10_NSNa 548.1566; found 548.1567.

### Cell culture:

HeLa and female mouse embryonic fibroblast (MEF) cells were cultured in DMEM low glucose media (Gibco, 10567022) supplemented with 10% FBS, penicillin (100 U/mL), and streptomycin (1 mg/mL) at 37 °C in a humidified incubator with 5% CO_2_. For experiments, cells were seeded at a density of 10k cells per well in a 4-well cover-slipped bottom slide, 100k cells/well in a 6 well tissue culture plate, and 500k cells in a 10 cm plate. Cells were allowed to settle overnight and then an appropriate amount of MM-JH-1 was added for the desired final concentration from a 100 mM stock. Alternatively, the equivalent volume of DMSO was added as a negative control.

### Cell Lysate collection:

After the appropriate time, cells were collected by trypsinization, counted, and centrifuged at 1000 *rpm* for 5 min in 1.5 mL Eppendorf tubes. Cells were lysed with RIPA lysis buffer on an ice bath for 10 min with occasional shaking and centrifuged at 4 °C with maximum for 10 min in 1.5 mL Eppendorf tube. Cell lysates were stored at −20 °C degrees for temporary storage or −80 °C for longer storage until use.

### CuAAC (Click) reaction:

#### With Cell Lysate (Using Click-iT kit C10276):

Up to 20 μL of lysate (up to 50 μg protein) was added to 100 μL Click-iT reaction buffer Component A (containing 40 μM of TAMRA-azide), 40 μL deionized water (to make final volume of 160 μL), 10 μL CuSO_4_, 10 μL Additive 1 solution, and after 2 min, 20 μL Additive 2 solution. Samples were mixed for 25 min in dark, after which time they were centrifuged to bring down the liquid. After labeling, the proteins were precipitated with methanol/chloroform. For MeOH/CHCl_3_, 600 μL MeOH, 150 μL CHCl3, and 400 μL water were added, and the samples mixed. Samples were centrifuged for 5 min at maximum speed and the top, aqueous layer removed and discarded. Next, 450 μL MeOH was added in two separate washes after which, the samples were centrifuged at maximum speed and the protein resided at the bottom of the tube. The resulted protein-pellet was air-dried (0.5–1 h).

#### With Fixed Cell (Using Click-iT kit C10269):

To the fixed and permeabilized cells a cocktail of 440 μL Click-iT reaction buffer (from stock of 1:10 diluted solution of component A), 10 μL CuSO_4_, 50 μL Click-iT cell buffer Additive (from stock of component C diluted in 4 mL of deionized water) and 2.5 μL fluorophore azide (from 1 mM stock solution of Alexa Fluor 488 azide or Oregon Green 488 azide in DMSO) was added. Samples were incubated in dark for 30 min at room temperature and washed three times with 1×PBS.

### Confocal microscopy:

Cells were grown to sub confluency, washed thrice with PBS, and fixed in 2% PFA in phosphate buffered saline (1×PBS) for 15 min at 37 °C in an incubator. Cells were washed thrice with PBS, and then permeabilized utilizing 0.1% Triton-X 100 in 1×PBS overnight at 4 °C. Cells were washed with 1×PBS and CuAAC labeling was performed with Alexa Fluor 488 azide or Oregon green 488 azide. Cells were washed thrice with PBS and blocked with 0.1% BSA in 1×PBSTw for 1 h. The cells were incubated with the primary antibody overnight and washed thrice with 1×PBSTw. They were then incubated with Alexa Fluor-conjugated secondary antibodies in 1×PBSTw for 1 h at room temperature. Cells were again washed thrice with 1×PBSTw. Samples were then mounted with DAPI and/or ConA Texas red. Fluorescence was detected with LSM 700 Confocal (Zeiss) using a laser to detect the stain and to detect the DAPI stain. A 63x water objective was used and samples were obtained using the same microscope settings for all samples within the same figure. Fluorescence intensity of images was analyzed with FIJI image processing software. Representative experiments are shown in the figures.

### Immunoblot:

Cell lysates were diluted to 1 mg/mL in RIPA buffer with a protease inhibitor cocktail and in appropriate volume of LDS loading buffer with β-mercaptoethanol (BME). To separate nuclear from cytoplasmic fractions we used the NE-PER kit (thermo fisher scientific). The samples were boiled for 10 min at 90 °C, and the protein was resolved by SDS PAGE: 4–12% Bis-tris gels (Invitrogen) with MOPS after which they were transferred to 0.2 um nitrocellulose membrane for 100 min at 100 V in transfer buffer. Blots were then blocked with Odyssey PBS blocking buffer for 1 h at room temperature and incubated with required primary antibodies at 4 °C for overnight. The next day blots were washed thrice with 1×PBSTw, incubated with appropriate secondary Odyssey antibodies at room temperature for 1 h, washed thrice with 1×PBSTw and developed under Odyssey instrument. Blots were quantified using the Image Studio software (Li-Cor).

### β-Elimination:

Cells were treated with the 100 μM MM-JH-1 and equivalent amount of DMSO as a negative control and incubated for 48 h. Then cells were collected by trypsinization and pelleted by centrifugation for 5 min at 1000 *rpm*, followed by washing twice with ice cold 1×PBS (1 mL), cell extracts were collected using RIPA buffer. Protein concentration was determined by BCA assay (Pierce, Thermo Fisher Scientific) and diluted to 1 mg/mL using RIPA buffer. Half of both DMSO and sample treated cell lysates were subjected to CuAAC reaction with TAMRA azide. Both the clicked, and not-clicked cell lysates were transferred onto nitrocellulose membranes. The blot was then washed once with 1×PBS for 10 min, and then incubated with H_2_O or 55 mM NaOH for 24 h at 40 °C. The blots were then washed thrice with 1×PBSTw, and then blocked with Odyssey PBS blocking buffer at room temperature for 1 h. The blots were then incubated with the appropriate primary antibody in blocking buffer with 0.1 % tween 20 for overnight at 4 °C. Both the anti-RL2 antibody (Thermo Fisher Scientific, MA1–072) and anti-TAMRA (Thermo Fisher Scientific, A6397) antibody was used at a 1:1000 dilution. The blots were then washed three times in 1×PBSTw for 10 min each, incubated with appropriate secondary Odyssey antibodies (1:10000 dilution) at room temperature for 1 h, washed thrice with 1×PBSTw and developed under odyssey instrument. Blots were quantified using Image Studio software (Li-Cor).

### PNGase F and Endo H treatment:

PNGase F (P0704S) and Endo H (P0702S) was obtained from New England BioLabs and treatments were performed according to the manufacturer’s protocol with some changes as described below. Cells were treated with the 100 μM of MM-JH-1 and equivalent amount of DMSO as a negative control and incubated for 48 h. Then cells were collected by trypsinization and pelleted by centrifugation for 5 min at 1000 *rpm*, followed by washing twice with ice cold 1×PBS (1 mL), cell extracts were collected using RIPA buffer. Protein concentration was determined by BCA assay (Pierce, Thermo Fisher Scientific) and diluted to 1 mg/mL using RIPA buffer. Both the DMSO treated, and sample treated cell lysates were subjected to enzyme treatment and water treatment as negative control. For PNGase F assay, to 10 μg of protein, 1 μL of 10×Glycoprotein Denaturing Buffer was added and incubated for 10 min at 100 °C. Then 2 μL of GlycoBuffer 2, 2 μL of 10% NP-40, 1 μL of PNGase F and 4 μL of deionized water (to make final volume of 20 μL), were added and incubated for 1 h at 37 °C. For Endo H assay, to the 10 μg of protein, 1 μL of 10×Glycoprotein Denaturing Buffer was added and incubated for 10 min at 100 °C. Then 2 μL of 10×GlycoBuffer 3, 1 μL of Endo H and 6 μL of deionized water (to make final volume of 20 μL), were added and incubated for 1 h at 37 °C. Enzyme (PNGase F and Endo H) treated, and water treated cell lysates were separately subjected to CuAAC reaction with TAMRA azide. The clicked, and not-clicked cell lysates were subjected to immunoblotting on nitrocellulose membrane and the blots were blocked with Odyssey PBS blocking buffer at room temperature for 1 h. The blots were then incubated with the appropriate primary antibody in blocking buffer for overnight at 4 °C. Both the anti-RL2 antibody (Thermo Fisher Scientific, MA1–072) and anti-TAMRA (Thermo Fisher Scientific, A6397) antibody was used at a 1:1000 dilution. The blots were then washed three times in 1×PBSTw for 10 min. incubated with appropriate secondary Odyssey antibodies (1:10000 dilution) at room temperature for 1 h, washed thrice with 1×PBSTw and developed under odyssey instrument. Then the blots were stripped using stripping buffer at 4 °C for overnight, washed and blocked with Odyssey PBS blocking buffer at room temperature for 1 h. The blots were then incubated with biotin conjugated Concanavalin A (Vector Lab, B-1005–5), diluted 1:1000 in PBS blocking buffer, for 1 h. The blot was then washed thrice with 1×PBSTw for 10 min each. Then the blot was incubated with IRDye^®^ 800 CW streptavidin Odyssey secondary antibody at 1:10000 in blocking buffer for 1 h. After being washed thrice with 1×PBSTw for 10 min each, the blot was developed using Odyssey instrument. Blots were quantified using Image Studio software (Li-Cor).

### Inhibitor Study:

HeLa cells were cultured as described earlier. For inhibitor studies, different concentrations of the inhibitors were added after 8 h of initial cell seeding and incubated for another 12 h. Cells were treated with the 100 μM of MM-JH-1 and incubated for 48 h. Either cell lysate was collected for immunoblotting or cells were fixed for confocal microscopic imaging.

### siRNA:

HeLa cells were cultured as described earlier. For siRNA experiment, DMEM (Gibco, A14430 without phenol red) was supplemented as described with or without antibiotics. The following siRNA reagents were purchased from Santa Cruz: transfection reagent (sc-29528), MGAT1 siRNA construct (human) (sc-40780), and Control siRNA-A (sc-37007). Lyophilized siRNA duplex was resuspended in 330 μL (MGAT1) or 66 μL (control) of RNase-free water provided. This yields a 10 μM solution in 10 μM Tris-HCl pH 8.0, 20 mM NaCl, 1 mM EDTA. Samples were aliquoted to 15 μL each and stored at −20 °C. HeLa cells were grown in DMEM media until experiment was performed. HeLa cells were seeded either in 6 well plates or in 4 well chambered slide in normal growth medium without P/S and grown to ~50% confluency in incubator at 37 °C, 5% CO_2_. On Day 1, for transfections, diluted 4 μL siRNA duplex (i.e., 0.5 μg or 50 pmols siRNA) into 100 μL siRNA transfection medium. Likewise, diluted 6 μL siRNA transfection reagent in 100 μL siRNA transfection medium. Added equal volumes of siRNA duplex and diluted transfection reagent and mixed gently. Incubated at room temperature for 45 min. Added 800 μL of siRNA transfection medium to each tube containing the transfection mixture. After washing cells 1x with siRNA transfection medium and aspirating the liquid, the duplex/transfection reagent mixture was added to the wells and cells were incubated for 8 h at 37 °C in the 5% CO_2_ incubator. After the incubation period, 1 mL normal growth medium was added, and cells were incubated overnight at 37 °C.

On Day 2, Day 1 procedure was repeated with one exception: when adding in the normal growth medium, DMSO or 100 μM 1,3-Pr_2_-6-OTs-GlcNAlk (MM-JH-1) was added to half of the cells. After 48 h of incubation, cells were either isolated by trypsinization and processed as described for immunoblot or fixed with PFA for confocal imaging.

### Glycomics analysis:

Cell pellets were washed with PBS buffer and lysed with lysis buffer containing 8 molar urea, to reduce viscosity sample were briefly sonicated using a microtip probe sonicator (Fisher Scientific). Lysate was centrifuged at 16,000 × *g* for 10 minutes at 4°C and the supernatant was buffer exchanged with 5 mM ammonium bicarbonate buffer to make 5 mM urea in final concentration using a 3 KD Amicon^®^ MWCO centrifugal filters (Millipore, Burlington, MA). Protein concentration was estimated with a Reducing Agent Compatible BCA Protein Assay Kit (Thermo Scientific^™^).

The *N*-linked glycans were released from 1000 μg of reduced and alkylated protein sample by treatment with Endo-H (NEB, P0702L, 50 U/μL) for 18 h at 37°C. The released *N*-glycans were collected by passing the digest through 10 KD Amicon^®^ MWCO *c*entrifugal filters (Millipore, Burlington, MA)) and dried by lyophilization.

The released *N*-linked glycans were permethylated using methods described elsewhere.^74^ The permethylated *N*-glycans were dissolved in methanol and mixed with an equal amount of DHB matrix solution (10 mg/ml in 1:1 methanol-water). The sample mixture was spotted on the MALDI plate, and MALDI-MS spectra were acquired in positive ion reflector mode on AB Sciex 5800 MALDI-TOF-TOF mass spectrometer.

### Biotin enrichment:

In T175 flask, HeLa cells were separately treated with 100 μM MM-JH-1 and incubated for 48 h. Cells were collected by trypsinization and pelleted by centrifugation (5 min, 1200 *rpm*), followed by washing with 1×PBS and cell extracts were collected using RIPA buffer. Protein concentration was determined by BCA assay (Pierce, Thermo Fisher Scientific). Cell lysates were subjected to CuAAC reaction with acid-cleavable Biotin DADPS picolyl azide (Sussex Research, BL000010). Pierce streptavidin magnetic beads (Thermo Fisher Scientific, 88817) were used to pull down biotin attached proteins. Initially streptavidin magnetic beads were pre-washed with binding/wash buffer (1×TBSTw) in a 1.5 mL microcentrifuge tube. Biotinylated proteins were added to the 1.5 mL microcentrifuge tube containing pre-washed magnetic beads and incubated at room temperature for 1 h with mixing. The magnetic beads were collected with a magnetic stand, washed twice with 1×TBSTw and IgG Elution buffer, pH 2.0 (Thermo Fisher Scientific, 21028). Resulting, collected protein was subjected to immunoblotting and Fibrillarin protein was detected with anti-fibrillarin nucleolar marker antibody (Abcam, ab5821).

### Statistics:

Graphpad (version 9) prism was used for all statistics. A two-way ANOVA was used to determine significance as indicated in figure legends. P values less than 0.05 were considered statistically significant. For confocal, quantification of the co-localization, Fiji (ImageJ v.1.52) was used with the co-localization plugin. The Pearson’s correlation coefficients between MM-JH-1 and fibrillarin or nucleolin were measured using three regions of interest (ROI) per image.
